# Modeling of emergency support capacity and optimization of delivery service system for urban living materials under uncertain situations: a case study of Xi’an City during COVID-19 epidemic

**DOI:** 10.1007/s43762-022-00076-5

**Published:** 2022-12-24

**Authors:** Jianpo Wang, Gang Li, Jiaobei Wang, Qifan Nie, Yue Yu, Tingting Xu

**Affiliations:** 1 College of Urban and Environmental Sciences, Northwest University, 710127 Xi’an, China; 2 Xi’an Public Security Bureau, 710000 Xi’an, China; 3 Shaanxi Key Laboratory of Earth Surface System and Environmental Carrying Capacity, Northwest University, 710127 Xi’an, China; 4 Department of Civil, Construction and Environmental Engineering, The University of Alabama, 35487 Tuscaloosa, AL USA

**Keywords:** COVID-19, Emergency support points, Express logistics terminal, Spatial accessibility, Emergency support and delivery system

## Abstract

The severe acute respiratory syndrome coronavirus 2 (COVID-19) pandemic has brought a heavy burden and severe challenges to the global economy and society, forcing different countries and regions to take various preventive and control measures ranging from normal operations to partial or complete lockdowns. Taking Xi’an city as an example, based on multisource POI data for the government’s vegetable storage delivery points, logistics terminal outlets, designated medical institutions, communities, etc., this paper uses the Gaussian two-step floating catchment area method (2SFCA) and other spatial analysis methods to analyze the spatial pattern of emergency support points (ESPs) and express logistics terminals in different situations. It then discusses construction and optimization strategies for urban emergency support and delivery service systems. The conclusions are as follows. (1) The ESPs are supported by large-scale chain supermarkets and fresh supermarkets, which are positively related to the population distribution.The spatial distribution of express logistics terminals is imbalanced, dense in the middle while sparse at the edges. 90% of express terminals are located within a 500 m distance of communities, however, some terminals are shared, which restrict their ability to provide emergency support to surrounding residents. (2) In general, accessibility increases as the number of ESPs increases; under normal traffic, as the distance threshold increases, the available ESPs increase but accessibility slightly decreases; with a traffic lockdown, the travel distance of residents is limited, and as ESPs increase, accessibility and the number of POIs covered significantly increase. (3) The spatial accessibility of the ESPs has a “dumbbell-shaped” distribution, with highest accessibility in the north and south, higher around the second ring road, slightly lower in the center, and lowest near the third ring road at east and west. (4) With the goal of “opening up the logistics artery and unblocking the distribution microcirculation”, based on “ESPs + couriers + express logistics terminals + residents”, this paper proposes to build and optimize the urban emergency support and delivery service system to improve the comprehensive ability of the city to cope with uncertain risks.

## Introduction

The novel severe acute respiratory syndrome coronavirus 2 (coronavirus disease 2019, COVID-19) is a major public health emergency with worldwide effects due to its strong infectious and pathogenic qualities (China National Health Commission, 2020). To block the transmission path of the virus and prevent contagion, some countries and regions with severe epidemics have been forced to take emergency measures, such as regional or national lockdowns. Societies and economies are confronting an uncertain situation that has a huge impact on the daily lives of residents (Sohrabi et al., 2020; Bai et al., 2020). Better meeting the needs of residents for living materials under this uncertain situation, especially ensuring the supply of necessities such as food, oil, meat and vegetables, has become an important task provided by urban emergency support. To avoid the insufficient supply of materials and nationwide rush to stockpile supplies during the epidemic, local governments often set up emergency support points (ESPs, for short) for centralized supply. At the same time, given the uncertainty of residential area lockdowns, national home isolation and traffic restrictions, an “online order + offline distribution” approach has gradually been adopted to guarantee supply and demand for residents, and express logistics are important to “the last kilometer” of delivery. Improvements to the emergency service system have become essential for the modernization of urban governance systems and governance capacity.

A series of studies on emergency support for public events such as infectious diseases have been carried out by scholars worldwide, including investigations into the spatial location and layout of emergency support sites and public service facilities, spatial accessibility, the layout and service of express logistics terminals, emergency logistics mechanisms and system platform construction. Balcik et al. (2008), Ngui et al. (2011), and Wu et al. (2010) adopted the perspective of urban geography and urban planning, which they combined with population distribution and land use to respectively study emergency rescue and shelter locations, develop planning layouts and model algorithms for different areas and evaluate and optimize the spatial layout based on different indicators and evaluation methods. Ekici et al. (2014) studied the food distribution during a possible influenza pandemic in the future, developed a disease transmission model, and combined it with facility locations and a resource distribution network model to find the approximate optimal solution for food distribution. The research achieved remarkable results and had a significant reference for the food security research under the current epidemic situation. In the context of the evolution of major infectious diseases, considering main factors such as space, information, materials, supply, demand and network, Ge and Liu (2020) applied Bayesian decision analysis, to model and analyze the emergency material allocation decision. Huang et al. (2010), Bell et al. (2013), Song et al. (2010), Tao et al. (2014), Fransen et al. (2015), Jiao et al (2021) and Mahyar et al (2021) based on the improved two-step floating catchment area method, carried out a series of studies on the spatial accessibility of public service facilities such as medical and education facilities, pensions, green space, grocery stores and healthcare facilities and analyzed differences in service in different regions. Li et al. (2018) and Huang (2017), based on the POI data of logistics express terminals, studied the form of support organization, location choice, spatial distribution characteristics, and spatial competition and cooperation among the group. Ernst (2003) analyzed and compared the differences between emergency logistics and traditional commercial logistics, focusing on the uncertainty related to emergency logistics, such as the uncertainty of merchants, locations and quantities involved in material supply and the complexity of transportation routes due to the impact of emergencies. Chen (2014) addressed the main problems existing in the construction of emergency logistics in China and offered optimization suggestions.

When confronting epidemics and other unusual situations, securing urban residents’ living materials establishes higher requirements for the emergency security network. From the macro perspective, it requires organization and coordination of the operational response process by government, society and multiple other actors, such as express logistics companies. At the same time, from the perspective of daily needs of residents, the accessibility of ESPs and quantity of food is very important. The above researches have made good progress in the respective fields, but there are few related and integrated researches at home and abroad at present. How to integrate the above research results, conduct exploratory analysis and research, build and improve the urban emergency distribution network service system, and improve the ability and efficiency of emergency security, has important practical significance.

This paper takes Xi’an city as the research area and focuses on urban emergency support under uncertain situations, based on data for Xi’an government’s reserved delivery points, population data and POI data for communities, hospitals, schools, express logistics, etc. Using kernel density estimation (KDE) and Gaussian two-step floating catchment area (G2SFCA) methods, this paper analyzes the spatial distribution of the Xi’an ESPs and different express logistics terminals and discusses the optimized layout and rational utilization for different situations. In addition, it also studies the urban emergency support and delivery service system based on “emergency support points + couriers + express logistics terminals + residents” to provide countermeasures and suggestions for the timely distribution of anti-epidemic materials, orderly circulation of residents’ living materials, and stability of the social order, aiming to serve as a reference for the construction of smart cities and resilient cities.

## Study area, data and methods

### Study area

As a comprehensive transportation hub and world-famous tourism destination, Xi’an has a large population and high density. At the end of 2018, the population was about 10 million, including 7.85 million in the main urban area, and the population density in the main urban area was about 1525 people/km^2^ (data from Xi’an statistical yearbook 2019). Before the establishment of the Xi’an epidemic prevention and control headquarters on January 23, 2020, over 1 million people flowed daily through Xi’an, of which over 70,000 were passing through Hubei before arriving in Xi’an, which put great pressure on epidemic prevention and control in Xi’an.

**Fig. 1:**
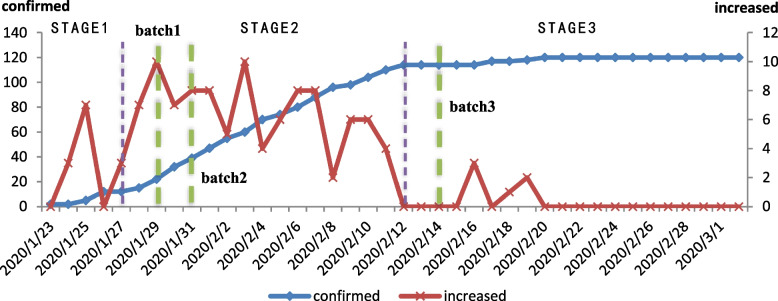
The evolution of the COVID-19 epidemic in Xi’an

**Fig. 2:**
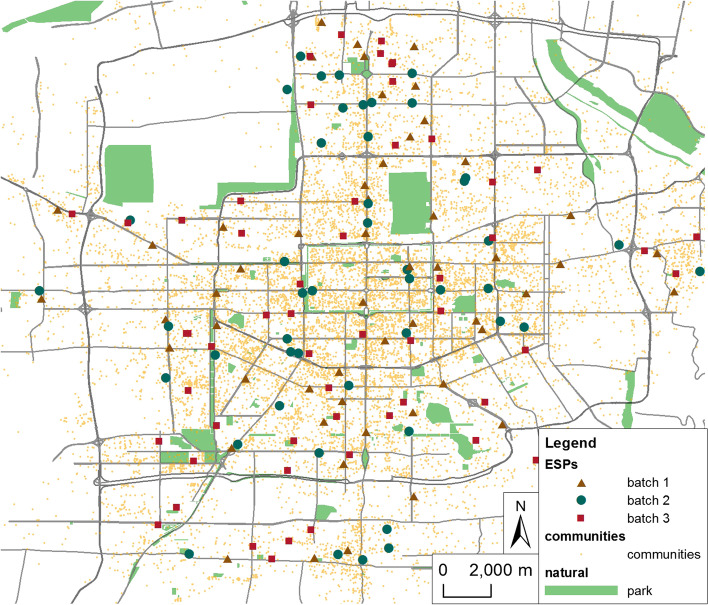
Distribution of governmental reserved vegetable delivery points and residential community points in Xi’an

Epidemic development in Xi’an can be divided into three stages: during the first ‘initial’ stage of epidemic development, from January 23 to January 27, a small number of confirmed cases gradually appeared; in the second ‘growth’ stage, from January 27 to February 12, the number of confirmed and suspected cases increased rapidly; in the third ‘stable’ stage, from February 12 to March 2, the number of confirmed cases became basically stable, and there have been few new cases since March 2 (Fig. 1). During the epidemic period, to ensure the continuous supply of necessities for citizens, the Xi’an municipal government, while taking step-by-step measures to upgrade their prevention and control abilities, established several government reserve vegetable delivery points as ESPs in 3 batches on January 29, January 31 and February 14 (Fig. 2).

Based on the above context, the main urban areas of Xi’an, such as Xincheng, Beilin, Weiyang, Yanta, Baqiao and Chang’an District, are taken as the main research areas. The geographic space range is E108.8°-E109.07° and N34.12°-N34.37°.

### Data

Covid-19 epidemic data of Xi’an City are taken from data reported by the Xi’an Health Committee (January 23 to March 2, 2020); the data of confirmed cases are collected and interpreted manually.

Xi’an municipal governmental reserved vegetable delivery point data originate from the relevant announcements of the Xi’an municipal government. Three batches of reserved vegetable delivery points were set up as ESPs, for a cumulative number of 58, 113 and 175 ESPs. The geographic location and relevant attribute information were collected for each delivery point.

POI data for Xi’an express logistics terminals, communities, designated medical institutions (fever clinics) and schools are all collected via webcrawler from the Gaode map. After February 20, 2020, the number of cases and communities subject to the epidemic in Xi’an stopped increasing. Therefore, after the epidemic entered the stable stage, February 24, 2020, was selected as the date for crawling, with a total of more than 20,000 items collected. The data preprocessing includes removing duplication, filtering, completing address information, and converting coordinates.

Xi’an population distribution data come from the census data of the Xi’an government, and the street level is selected as the spatial statistical unit. Xi’an administrative division and traffic network data are from OpenStreetMap, based on which the Xi’an road topological network with the network path topological relationship is established.

### Methods

This paper adopts spatial accessibility analysis based on the Gaussian two-step floating catchment area method (2SFCA), kernel density estimation (KDE), buffer analysis, spatial correlation analysis and other spatial analysis methods.

1. points k within search radius

Spatial accessibility is affected by many factors, including supply point scale, demand point scale and travel boundary factors. The two-step floating catchment area method was first proposed by Radke and Mu (2000) and further improved by Wang and Luo (2005). Because 2SFCA (and its extended form) considers various factors, it is widely used in accessibility measurements for various facilities. It takes the supply point and the demand point as the center of the mobile search and sets a clear critical range (search radius ${d}_{0}$). The calculation results are determined by the supply and demand of the supply and demand points and are not limited to the administrative boundary. The basic idea is as follows:

- ①For each supply point j, search all the demand points k within search radius ${d}_{0}$ and calculate the supply–demand ratio ${R}_{j}$;
- ②For each demand point i, search all the supply points j within search radius${d}_{0}$ and sum all the supply–demand ratios ${R}_{j}$ to obtain the accessibility ${A}_{i}^{F}$ of point i.

The calculation formula is as follows:

1 $$A_i^F=\sum_{j\in\left\{d_{ij}\leq d_0\right\}}R_j=\sum_{j\in\left\{d_{ij}\leq d_0\right\}}\left\{\frac{S_j}{\sum_{k\in\left\{d_{ij}\leq d_0\right\}}D_k}\right\}$$

In the formula, i is the demand point; j is the supply point; ${A}_{i}^{F}$is the accessibility of demand point i; ${d}_{ij}$ is the cost of transporting supplies between demand point i and supply point j, generally measured as distance or time; ${R}_{j}$is the ratio of the facility scale of supply point j to the population served within search radius ${d}_{0}$; ${S}_{j}$ is the supply scale of supply point j; and ${D}_{k}$ is the demand scale of demand point k.

(2) Gaussian 2SFCA(G2SFCA)

Extensions of the 2SFCA can be divided into four types: extension of distance decay function, extension of the search radius, extension of the demand or supply competition, and extension based on travel mode (Tao and Cheng, 2016). An additional distance decay function within the search radius of 2SFCA is added in the first extension. To obtain this kind of extension, Wang (2012) proposed a general form of the 2SFCA (generalized 2SFCA) and added a distance decay function$f\left({d}_{ij}\right)$ to the model to summarize and express the distance decay function forms in different extensions:

2 $${\text{A}}_i=\sum_{j=1}^n\frac{S_jf\left(d_{ij}\right)}{\sum_{k=1}^mD_kf\left(d_{kj}\right)}$$

Where${ \text{A}}_{i}$ is the accessibility score of demand point i, which is the average accessible facility resources of each demander at demand point i; $f\left({d}_{ij}\right)$ is a general distance decay function; and the other variables have the same meaning as in formula (1). Based on the decay trend, the distance decay function can be divided into several forms, such as segmented jump, gravity, kernel density and Gaussian. The Gaussian 2SFCA shows “S” type decay, and accessibility is slower in the near and far stages given the distance decay speed but faster in the middle stage. In this paper, the Gaussian 2SFCA is selected to evaluate spatial accessibility.

## Analysis on emergency support and distribution service of urban living materials

Under normal circumstances, most of the residents mainly drive or walk to the supermarkets, vegetable wholesale markets and convenience stores to purchase daily necessities. The “new retail” stores, represented by Hema Fresh, have gradually become a common way to guarantee the supply and demand of residents through the consumption mode of online ordering and offline distribution. Based on market means, this kind of urban living materials distribution service system can better meet the daily needs of living materials of residents normally. With the spread of the epidemic and the change in the number of infected people, different countries and regions have implemented different levels of preventive and control measures based on their national condition, cultural traditions and other context-specific factors to control the operation or flow of cities, communities, populations, and traffic. Affected by the government’s emergency measures, the material supply of wholesale markets and supermarkets, express logistics distribution would be inevitably restricted, which brings about a variety of uncertain situations, and the material security service system is bound to be weakened to varying degrees.

**Fig. 3:**
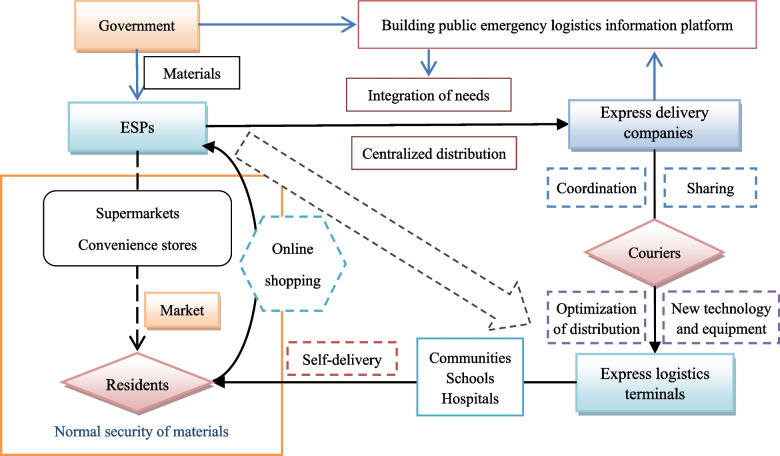
Emergency support and distribution system of urban living materials based on “ESPs + couriers + express logistics terminals + residents”

Logistics network system is connected with production and life, “opening up the logistics artery and unblocking the distribution microcirculation”, is the basic requirement and responsibility of urban emergency material support under the epidemic situation. Under the epidemic situation, Xi’an municipal government has set up a number of ESPs for centralized supply of urban living materials in batches, and the security points are allowed to purchase on-site or online. In the case of limited purchase, residents purchase daily necessities through e-mall according to personal or family needs. The couriers pick up materials according to the order and deliver them to the express terminals (pick-up points) or directly deliver them to the residents. The terminal outlets of express logistics play an important role in ensuring “the last kilometer” and focus on serving the residents within a certain range. In view of the above special circumstances, this paper proposes an emergency support and distribution system of urban living materials based on “ESPs + couriers + express logistics terminals + residents”, as shown in Fig. 3.

In this system, the four “roles” play different roles, so the impact on emergency support and distribution services is also different. ESPs generally rely on large and medium-sized supermarkets. The storage capacity and transportation convenience level of supermarkets and the spatial layout of ESPs in the city directly affect the material supply capacity, coverage and quality difference. The number of couriers and the spatial layout of express logistics terminal network affect the ability of material distribution. The spatial distribution of residents affects the distribution of material demand correspondingly. This paper focuses on urban ESPs and express logistics terminals, and then discusses the evaluation and improvement of service capability of material emergency support and distribution system.

## Spatial patterns of ESPs and express logistics terminals

### Dependent objects and distribution of ESPs

With the continuous increase in confirmed cases and affected areas during the epidemic, to enhance its material support capacity, the Xi’an municipal government established three batches of reserved vegetable delivery points as ESPs relying on supermarkets and convenience stores, numbering 58, 55 and 62 (Table 1), of which 54, 47 and 56 are included in the study area of this paper (Fig. 2).

**Table 1:** Brands and quantity of different batches of ESPs

ESPs	Batches			
Brands	Batch1(Jan.29)	Batch2(Jan.31)	Batch3(Feb.14)	Percent
CR Vanguard	37			23.6%
Chengshan Farm	9			5.7%
Mihe Fresh	8			5.1%
RENRENLE		28		17.8%
Lotus		5		3.2%
Wal Mart		4		2.5%
Hema Fresh		3		1.9%
Jianjun		2		1.3%
Hongye Life		1		0.6%
Juyou Fresh		1		0.6%
Leran Fresh		1		0.6%
Xi’an Xinbeicheng		1		0.6%
Smart Home		1		0.6%
Yonghui			48	30.6%
Keyouduo			4	2.5%
7Fresh			2	1.3%
Furong Xingsheng			1	0.6%
Qimu Fresh			1	0.6%
Total	54	47	56	157

The main dependent objects of the ESPs are Yonghui (48), CR Vanguard (37), RENRENLE (28), Lotus (5), Wal Mart (4) and other large-scale chain supermarkets, in total accounting for 77.7%; Chengshan Farm (9), Mihe Fresh (8), Hema Fresh (3), 7Fresh (2) and other fresh supermarkets, accounting for 15.9%; and other supermarkets accounting for 6.4%. As ESPs, large supermarkets offer basic advantages: abundant materials, free choice, low price, convenient shopping and excellent service make this format the pillar of the retail market. Based on a highly integrated online and offline business model and an excellent logistics system, “New Retail”, represented by Hema Fresh and 7Fresh, plays a prominent role during the epidemic.

The ESP is mainly responsible for the supply of materials to surrounding communities within a certain range, and when selecting the site, a large reserve capacity and good transportation facilities are generally needed. The layout of large supermarkets and “new retail” is affected by many factors, including population, transportation, business environment and urban planning. Population distribution and network layout reinforce each other, and “network follows population” is the main feature of their relationship (Dong, 2012). Strong storage and logistics capacity, convenient transportation, familiar geographical location, large-scale and high-density surrounding communities, etc., all contribute to the ability to quickly guarantee emergency materials based on the largest capacity of large and medium-sized supermarkets. The spatial distribution of large supermarkets in Xi’an is imbalanced. Large supermarkets and chain supermarkets are mainly concentrated in the central business district of the city and less concentrated in the suburbs and secondary business centers. The dense network in the center of the city results in a small radius for the supermarket business circle, which intensifies industry competition and results in wasted materials and personnel. Simultaneously, residents in the newly built areas and suburbs must travel long distances to the city center to buy daily necessities, which creates inconvenience in their daily lives (Zhou, 2011).

### Spatial pattern of express logistics terminals

The terminal is the last link of the express logistics supply chain. As the beginning and end of the city’s express logistics network, the express logistics terminal network is directly aimed at solving the “last kilometer” distribution bottleneck. The layout of express logistics terminals reflects the overall plan considering the number, scale, service radius, and spatial location of express enterprise outlets. Influenced by the road system, cost, natural environment, demand market and other practical factors, it generally follows the principles of supply–demand balance, strategic consistency, maximization of benefits and location optimization (Song, 2017).

**Fig. 4:**
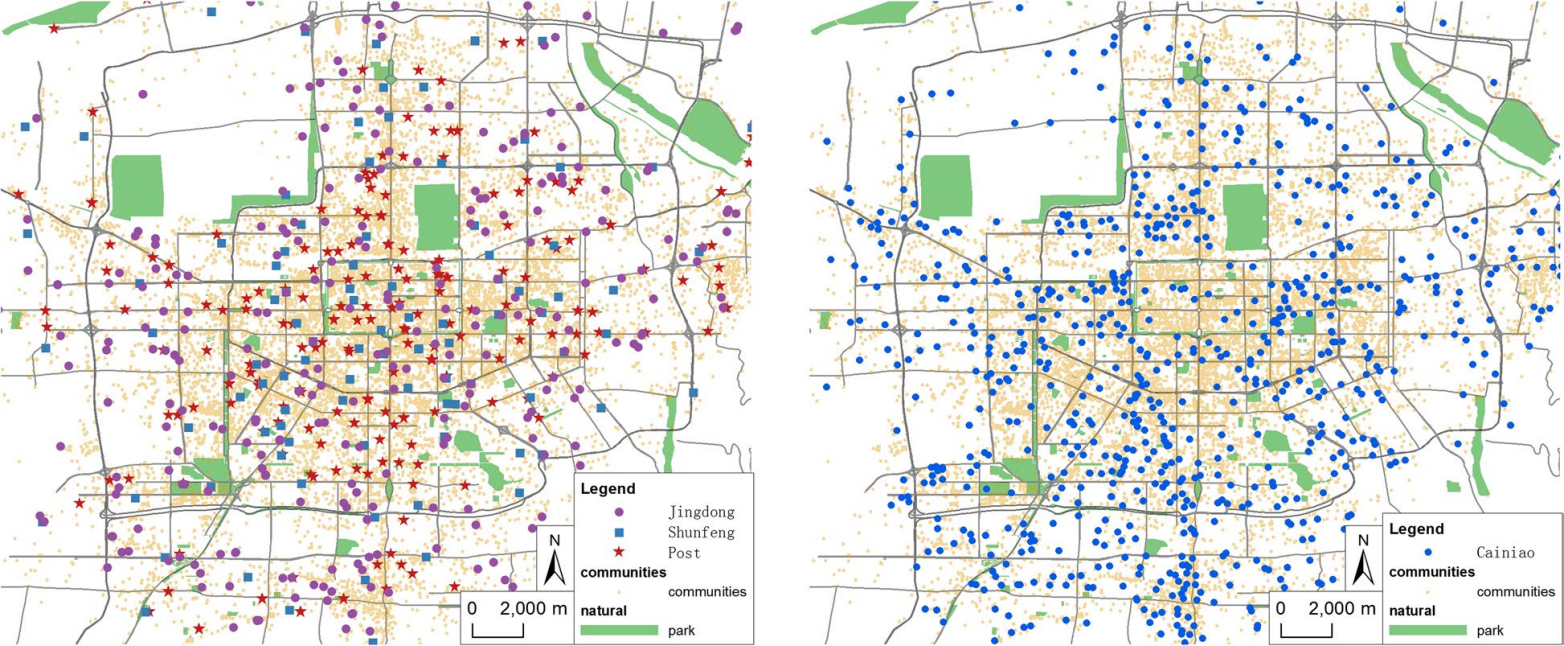
The network layout for Post, Shunfeng, Jingdong and Cainiao in Xi’an

China Post (hereinafter referred to as Post), Shunfeng Express (hereinafter referred to as Shunfeng), Jingdong Logistics (hereinafter referred to as Jingdong) and Cainiao post station (hereinafter referred to as Cainiao) have played a particularly prominent role in this epidemic. This paper mainly studies the four types of express logistics terminals (including self-reference points) (Fig. 4). Post is a government-oriented and state-owned enterprise with hierarchical network characteristics. Its stations are generally branch service outlets of China Post, such as post offices and convenient service stations. Cainiao post station is a market-oriented and atomic logistics service platform. It is committed to providing diversified last kilometer services, with a large number of outlets, mainly for communities and schools. Shunfeng, Jingdong, etc. also provide a variety of service forms, including outlets, convenience stores, cooperative self-delivery points, etc.

First, the street-level population density data of Xi’an is rasterized, and based on the “kernel density analysis” tool of ArcGIS, the kernel density distribution grid data are generated for the community, Post, Shunfeng, Jingdong and Cainiao POI data. Based on the “multivariate\band collection statistics” in the “spatial analyst tools” tool of ArcGIS, a correlation analysis for 6 grid layers is performed, and the correlation matrix is shown in Table 2.

**Table 2:** Spatial correlation analysis of population density, communities and various express logistics terminals

POI	Population density	Communities	Post	Shunfeng	Jingdong	Cainiao
Population density	1	0.89245	0.78182	0.623	0.53278	0.75495
Communities	0.89245	1	0.84726	0.64927	0.54911	0.8
Post	0.78182	0.84726	1	0.52825	0.41236	0.64175
Shunfeng	0.623	0.64927	0.52825	1	0.41154	0.46635
Jingdong	0.53278	0.54911	0.41236	0.41154	1	0.55992
Cainiao	0.75495	0.8	0.64175	0.46635	0.55992	1

Compared with Fig. 7 and Table 2, the overall characteristics of express terminals in Xi’an are found as follows:

1. The spatial distribution is not balanced, and the overall distribution pattern is “dense in the middle and sparse on the side”. The distribution in the second ring is relatively dense, and the center of gravity is located in the middle and southwest of Xi’an. The Cainiao, Post and Shunfeng outlets gradually and obviously become increasingly sparse from the inside to the outside, while the distribution of Jingdong outlets is relatively more uniform.
2. The spatial distribution of terminals has a strong positive correlation with the population distribution. Based on the data for street-level population density, a correlation analysis of communities, Post, Shunfeng, Jingdong and Cainiao, is conducted. The correlation coefficient between communities and population is 0.89245, which shows that although there are some differences between their distributions, they are basically the same. Post, Cainiao and the population distribution have a stronger correlation, which shows that the layout of their network is greatly affected by the distribution of communities, which helps them to provide better quality services for the surrounding residents. Cainiao is dedicated to providing “last kilometer” post station service for users from communities and schools. To facilitate self-service by residents, the Cainiao terminal is usually located near the community entrance and exit, so its layout is closer to the community. Jingdong and Shunfeng mainly provide door-to-door service, so the correlation with population distribution is weak.
3. All types of express logistics terminals have a positive correlation with each other, indicating that there is cooperation within the competition between them, aiming at mutual benefit and win-win results that provide better services for users.

### Analysis of logistics terminals integration in an emergency

Although all kinds of express logistics terminals cover the urban area of Xi’an, they each have their own emphasis. Under emergency conditions, the number of express deliveries in communities increased dramatically. It is very important to improve their service and security capacity by integrating, realizing shared services, discovering and filling the gaps between terminals. The closer the terminal network is to the community, the greater the number and the more sufficient the security provided to the community residents.

This paper integrates all of the collected express logistics terminals and analyzes the buffer zones of 13,592 POIs for communities, schools and hospitals collected in Xi’an at different distances (Table 3). Within “the last kilometer”, 99% of POIs are covered, which indicates that Xi’an express logistics is in good condition and can basically meet the daily life needs of residents. The coverage rate within 300 m is 67%, and within 500 m is 90%, indicating that most express outlets are located within 500 m of the community, which offers good access for residents on foot.

**Table 3:** The number of POIs covered by express logistics terminals at different distances

Distance(m)	100	200	300	400	500	600	700	800	900	1000
Covered POIs	2224	6060	9136	11,236	12,288	12,831	13,125	13,275	13,369	13,441
Percent	16%	45%	67%	83%	90%	94%	97%	98%	98%	99%

**Fig. 5:**
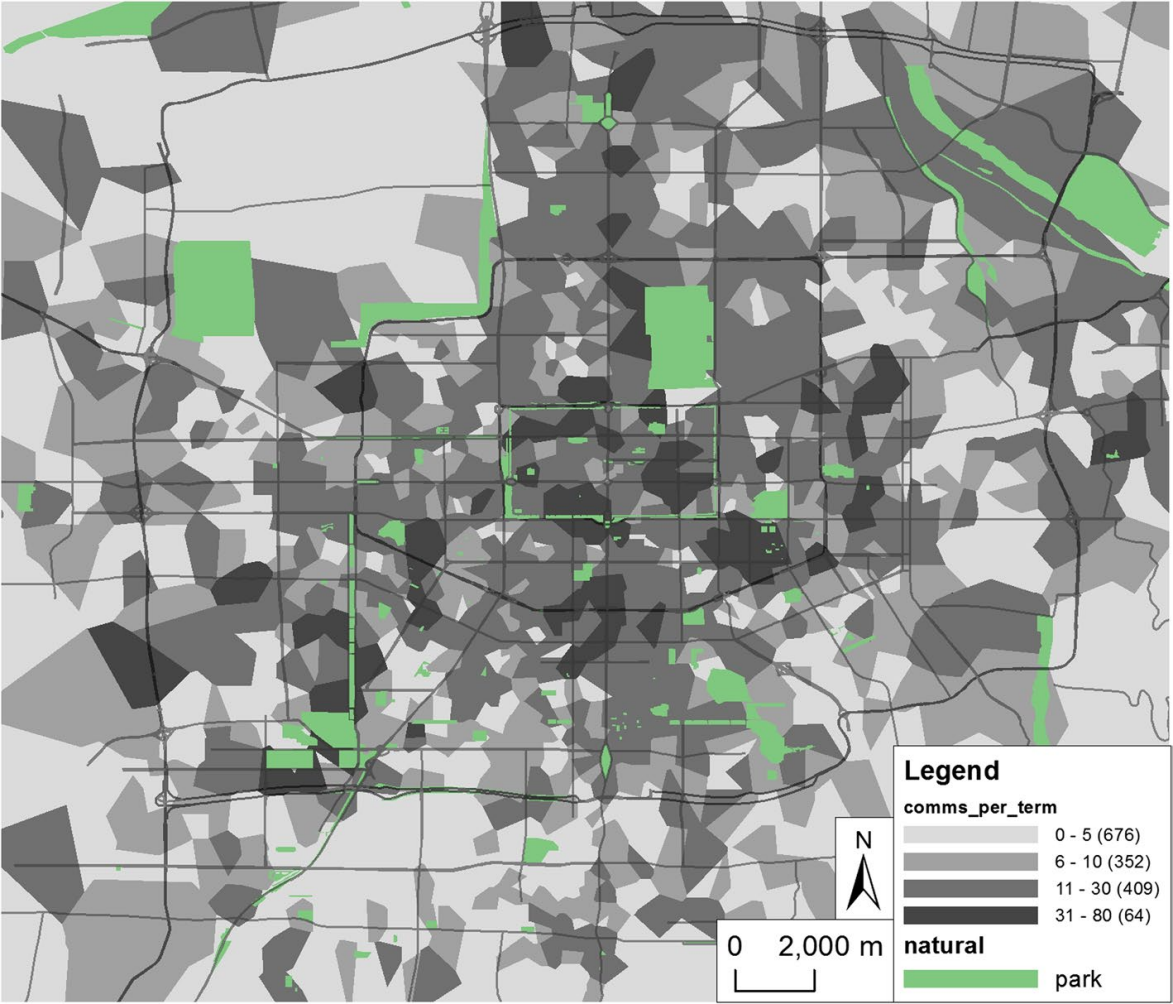
Service analysis of express logistics terminals to communities

Generally, there are one or more express outlets with good accessibility near each community, but there are also many residential areas sharing one or more express outlets. Thiessen polygon is generated for all express terminals in Xi’an, and the number of communities within each polygon can be estimated as the number of communities served by each express terminal. As shown in Fig. 5, the label value is the number interval of communities served by terminals, and the numbers in brackets are the number of express terminals in this interval. It is found that all the express terminals in the north and south areas between the second ring road and the third ring road basically serve more than 10 communities. Although the density of express terminals in this area is relatively high, the communities are also dense.

Under the epidemic, there are contradictions between the rapid growth of express businesses, the space capacity of express outlets and the strength of logistics practitioners (i.e., network contract distributors), which will have negative effects on the service guarantee of express outlets and the safety of residents visiting these terminals.

## Analysis of emergency distribution system based on spatial accessibility

### Spatial accessibility analysis of POIs in different situations

**Fig. 6:**
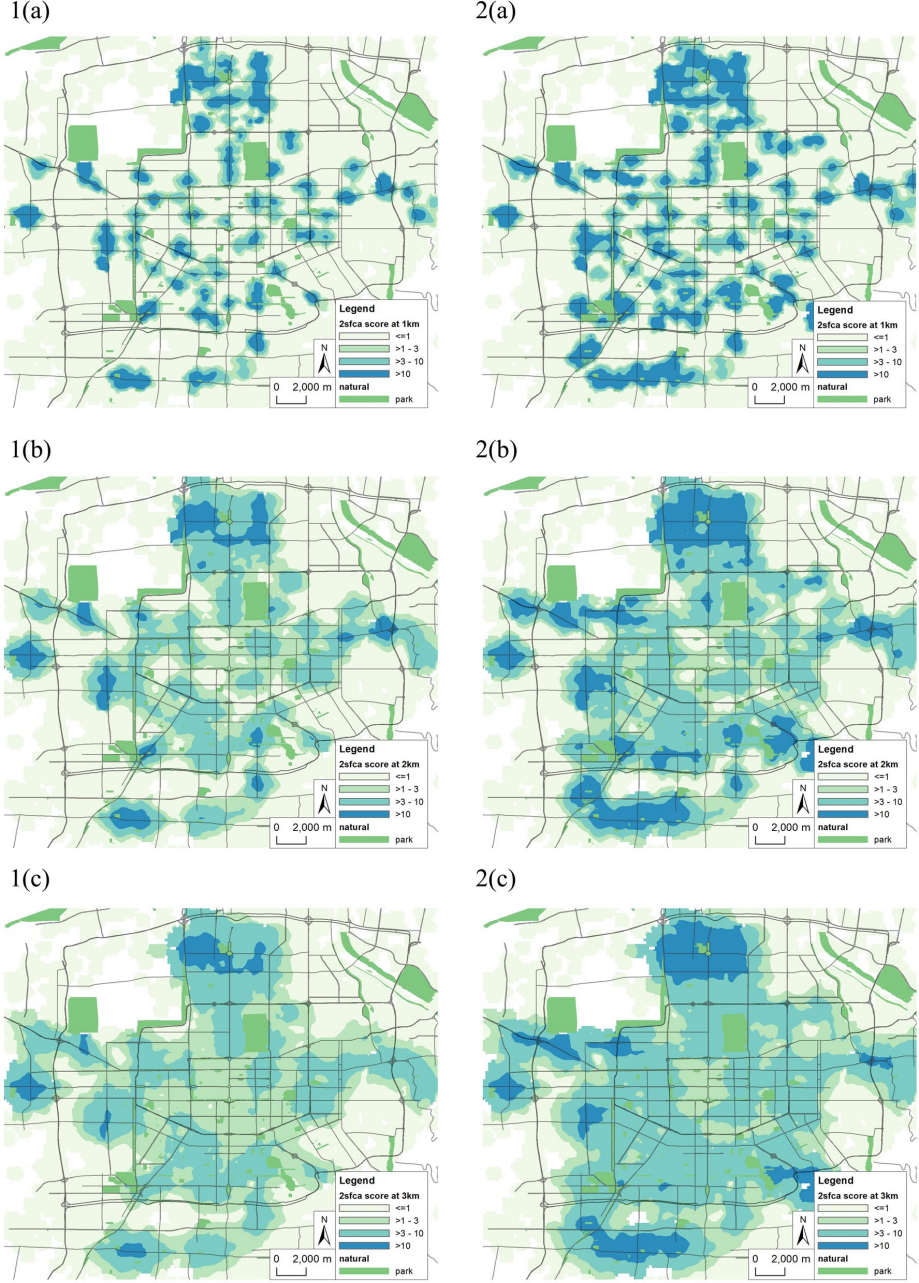
Accessibility analysis of two batches of ESPs with different distances of 1000, 2000, 3000 m

As mentioned above, the spatial accessibility is mainly affected by such factors as supply point scale, demand point scale and travel distance. The supply capacity of ESPs relying on large chain supermarkets, fresh supermarkets and other supermarkets are set to be 500 000, 200 000,100 000 person separately. Since the specific population of each residential area cannot be obtained temporarily, to simplify processing, based on the total population of Xi’an and the number of residential areas, the population of each residential area is set as 1000. Different levels of urban traffic lockdown affect residents’ travel mode and thereby affect residents’ willingness to travel certain distances. Given a traffic lockdown, residents mainly walk, and the distance traveled is limited to generally no more than 1000 m. Under normal traffic conditions, the travel distance of residents increases. Different distance thresholds of 1000 m, 2000 and 3000 m are set, and based on the Gaussian 2SFCA, a spatial accessibility analysis is carried out for 13,592 POIs representing communities, schools, hospitals, and the first and second batches of ESPs in Xi’an City (due to the close time interval between the two, the calculation is combined), and all ESPs after the third batch are then added, and interpolate the accessibility values in Kriging. The visualization results are shown in Fig. 6.

Comparing different quantity of ESPs, the results show that (1) By increasing the number of ESPs, the accessibility of POIs are greatly improved under different distance thresholds; (2) within small distance thresholds, an increase in the number of ESPs makes them accessible to more POIs such as communities; this indicates that if a traffic lockdown occurs, the number of ESPs should increase as much as possible to better guarantee the supply of living materials to residents; and (3) as the distance threshold increases, POIs such as residential areas have more choices for ESPs, and the coverage of ESPs increases, but the overall accessibility of POIs within coverage area decreases slightly, indicating that support services are more decentralized, which is conducive to improving the fairness and efficiency of emergency security of social materials.

Comparing different distance thresholds, the analysis results (Fig. 6) show that the accessibility has a “dumbbell-shaped” distribution in the north-south direction. Weiyang District in the north and Chang’an District in the south have the highest accessibility, the accessibility around the second ring road is higher, inside the city wall is slightly lower, and near the third ring road in the east and west is the lowest.This structure is generally coordinated with the population distribution of Xi’an City and also related to its long-term development plan. Since the founding of the People’s Republic of China, Xi’an city has developed in three directions: east, west and south. The eastern, western and southwestern suburbs are mainly industrial areas, and cultural, educational and scientific research units are concentrated in the southern suburbs. Therefore, the population in Xi’an city is most dense inside the city wall, followed by the southwest and east. In the 20th century, with municipal government offices moving to the north and the development of the northern suburbs, the population in the north has gradually increased, but there is a certain population concentration on both sides of Metro Line 2 that is confirmed in the layout of ESPs. In the surrounding areas of Xi’an City, there are relatively few support points and relatively long support distances; this is especially true in the Chanba area in the northeast and Qujiang area Phase II in the southeast, which have experienced rapid economic and population development in recent years.

### Spatial analysis of ESPs and express logistics terminals

**Fig. 7:**
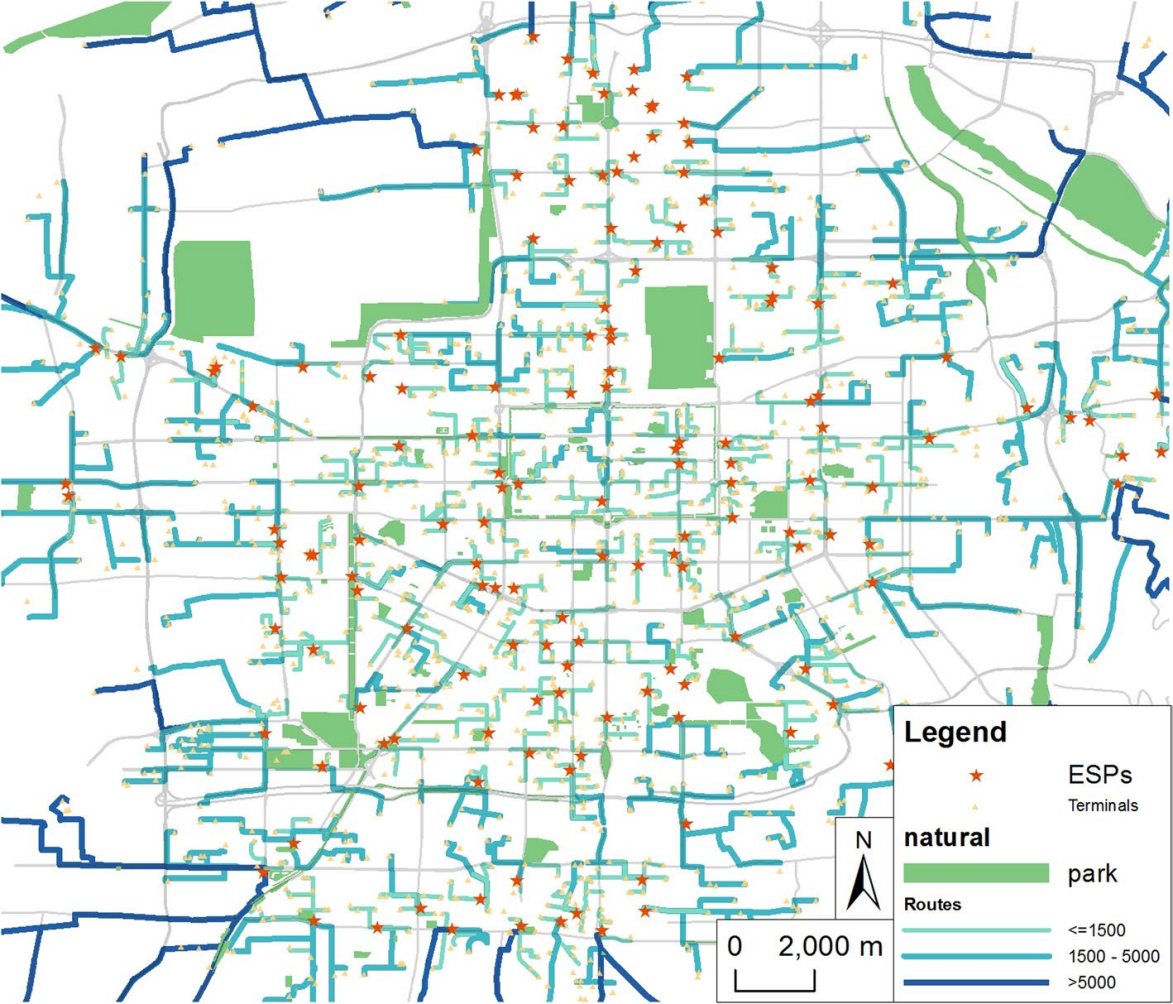
Shortest path analysis of terminals and ESPs

Based on the OD cost matrix of road network analysis, the shortest path from the ESPs to the express logistics terminals is constructed (Fig. 7). The result shows that the overall accessibility decreases from the center to the outside, with the central and southern part of the city being the center. The average length of the support route from each ESP to each terminal network is 2071 m, and the length of the support route in the south-north direction of the second and the third ring roads is basically within 1500 m, consistent with the densely populated area of Xi’an city; the support route in the east and west side of the third ring road and Chang’an District is slightly longer, but mostly within 5000 m; and there is a large length of the support route in the four corners of Xi’an city, which is related to the sparse distribution and low population density in these regions.

## Existing problems and countermeasures

In the context of an epidemic, the logistics network plays an important role in ensuring the smooth connection of the industrial chain and a stable supply of residents’ necessities, providing logistics support to promote economic development and meet people’s life needs. However, it also exposes existing difficulties and shortcomings. (1) During the Spring Festival, some employees return home, the logistics efficiency of enterprises decreases, and some terminals are forced to close due to lack of staffing; (2) the number of express deliveries increases in a context of closed communities and traffic lockdown, but the space and manpower of terminals restrict their ability to guarantee service; (3) the logistics industry has been small, scattered and disorderly for a long time due to a lack of overall planning and awareness of sharing and cooperation; (4) affected by the traffic lockdown under the epidemic, the supply channel between the suppliers and the consumers of materials (especially vegetables and fruits in the city suburbs) has been interrupted, resulting in a disconnection between supply and demand. Although internet-based express logistics has played a role in opening up the supply chain between producers and consumers, it still needs further guidance, communication and optimization.

In view of the above problems, suggestions are proposed considering different levels and dimensions, such as government, retail logistics industry and new technology application:

1. The government should strengthen urban planning to ensure the implementation of clear measures and responsibilities. It needs to deepen the planning concept for resilient cities and strengthen the ability to resist risks and self-regulate. As the unit of the city, the planning of the daily lifecycle of the community should consider uncertain situations such as epidemics and disasters and actions such as improving the accessibility of ESPs and medical and living facilities. An epidemic prevention and control plan and emergency response plan should be formulated and implemented to better define the leadership and responsibilities of each department, allowing them to effectively carry out various types of rescue work. Knowledge about epidemic prevention and control must be popularized and various resources and forces of society guided and integrated, such as enterprises, institutions, community autonomous organizations, community volunteers, etc. Government focus should be on strengthening support for the express logistics industry, coordinating cooperation between different enterprises, providing convenient measures for the road transportation segment of logistics, carrying out precise control over the end section of express delivery, and avoiding a “one size fits all” solution. At the same time, government must actively promote the integration of intelligent terminal facilities and public service terminal stations into the construction of urban and rural public infrastructure, build intensive and shared public service terminals, and accelerate the completion of the short board of the express infrastructure.
2. The operation mode and service system based on “e-commerce + logistics” have shown great advantages in this epidemic. Strengthening cooperation between the retail industry and logistics enterprises is the appropriate direction for future development, as it will improve the integration and coordination of the industrial chain and supply chain operations and enhance risk response and emergency support capabilities. First, the retail and logistics industry network layout should be reasonably planned, and the number of internal network points in the same region should be optimized in combination with urban planning, population distribution and road traffic. Second, cooperation and coordination mechanisms should be established with logistics enterprises. Integrating logistics enterprises, improving the network scale advantage and strengthening the ability to minimize risk will be conducive to the long-term development of the logistics industry. Establishing cooperative mechanisms such as warehouse sharing, personnel sharing and data sharing can maximize the effectiveness of these enterprises. By sharing warehousing and employees, material supply and prompt delivery is guaranteed for the coverage area. Data sharing can be used to integrate the material supply chain and logistics distribution system to realize network sharing and complementary advantages.
3. New technologies and intelligent facilities represented by artificial intelligence, big data, cloud services, the Internet of Things and regional chains play an important supporting role in epidemic monitoring and analysis; virus tracing, prevention and treatment; resource allocation, etc. but the application of new technologies and new products is still in the early stage; the combination of social governance and industry practice has wide future application prospects for building smart cities. In addition, the new demands for intelligence and contact-free interaction for the logistics industry and the application of intelligent logistics equipment such as UAV automatic sorting are also expected to accelerate the deep integration of logistics activities and new technologies as well as the construction of intelligent logistics.

## Conclusion and prospects

### Conclusion

Taking Xi’an city as an example and utilizing multisource POI data for government vegetable storage delivery points, logistics terminal outlets, designated medical institutions, communities, etc., this paper applies the Gaussian two-step floating catchment area method (G2SFCA) and other spatial analysis methods to analyze the spatial layout of ESPs and express logistics terminals in different situations; based on the results, it then discusses the construction and optimization of an urban emergency logistics network service system based on “ESPs + couriers + express logistics terminals + residents”. The results show the following.

1. In the existing emergency support system, the ESPs mainly rely on large-scale chain supermarkets in the central city and small and medium-sized supermarkets or convenience stores in the suburbs and secondary business center areas. In the surrounding areas, especially in the northeastern and southeastern areas with rapid economic and population development, the layout of ESPs is relatively insufficient.
2. The spatial distribution of express logistics terminals in Xi’an is not balanced, and the overall pattern is “dense in the middle and sparse on the side”. The distribution in the second ring is relatively dense, and the center of gravity is located in the middle and southwest. The correlation with the population distribution was the strongest for Post and Cainiao, but weaker for Jingdong and Shunfeng. 90% of express terminals are located within a 500 m distance of communities, however, some terminals are shared, which restrict their ability to provide emergency support to surrounding residents.
3. In general, accessibility increases as the number of ESPs increases; under normal traffic, as the distance threshold increases, the available ESPs increase but accessibility slightly decreases; with a traffic lockdown, the travel distance of residents is limited, and as ESPs increase, accessibility and the number of POIs covered significantly increase.
4. The spatial accessibility of the ESPs has a “dumbbell-shaped” distribution, with highest accessibility in the north and south, higher around the second ring road, slightly lower in the center, and lowest near the third ring road at east and west.
5. The urban emergency logistics network service system is constructed and optimized with the goal of “opening up the logistics artery and unblocking distribution microcirculation”, based on “ESPs + couriers + express logistics terminals + residents”. The government and the retail and logistics industries should give full play to their respective responsibilities and missions and increase the application of new technologies and cooperation to improve the city’s ability to respond, adapt and recover in the face of uncertain risk factors and enhance the city’s resilience.

### Prospects

Due to the limited research period and data obtained, there are still some weaknesses in this study: the demand for living materials in different communities should be in direct proportion to the population; supermarkets and express terminals have differences due to their service areas and the population they serve; and some terminals are closed during the Spring Festival. Due to the lack of such data, this paper has simplified the analysis process. In the next step, more relevant data should be collected to deepen the investigation, such as the population of the communities and the service ability of different supermarkets, express terminals, etc. Targeted in-depth research should be carried out for different types of express terminals, such as post stations, convenience stores, storage facilities, and transfer stations, to provide a reference and support for the optimization of the urban emergency logistics network service system, offer suggestions for the rapid supply of emergency materials and for the planning and construction of resilient cities in different contexts, further promote the national public health emergency management system, and improve the city’s ability to respond to major public health emergencies.   

## Data Availability

All the data in this paper are from the government public website.
